# Impact of Varying Sleep Pressure on Daytime Sleep Propensity in Healthy Young and Older Adults

**DOI:** 10.3390/clockssleep7010002

**Published:** 2025-01-02

**Authors:** Stella de Haan, Marine Dourte, Michele Deantoni, Mathilde Reyt, Marion Baillet, Christian Berthomier, Vincenzo Muto, Gregory Hammad, Christian Cajochen, Carolin F. Reichert, Micheline Maire, Christina Schmidt, Svetlana Postnova

**Affiliations:** 1 Sleep and Chronobiology Laboratory, GIGA-CRC Human Imaging, University of Liège, 4000 Liège, Belgium; sdehaan@uliege.be (S.d.H.); marine.dourte@uliege.be (M.D.); michele.deantoni.md@gmail.com (M.D.); mathilde.reyt@gmail.com (M.R.); vincenzo.muto@uliege.be (V.M.); gregory.hammad@uliege.be (G.H.); 2 Psychology and Neurosciences of Cognition Research Unit (PsyNCog), Faculty of Psychology and Educational Sciences, University of Liège, 4000 Liège, Belgium; 3 Athinoula A. Martinos Center for Biomedical Imaging, Department of Radiology, Massachusetts General Hospital and Harvard Medical School, Boston, MA 02115, USA; 4 Physip S.A., 6 Rue Gobert, 75011 Paris, France; c.berthomier@physip.fr; 5 Human Chronobiology and Sleep, University of Surrey, Guildford GU2 7XH, UK; 6 Centre for Chronobiology, Psychiatric Hospital of the University of Basel, 4002 Basel, Switzerland; christian.cajochen@upk.ch (C.C.); carolin.reichert@upk.ch (C.F.R.); mi_maire@hotmail.com (M.M.); 7 Research Cluster Molecular and Cognitive Neurosciences, University of Basel, 4055 Basel, Switzerland; 8 Circadian Physics Group, School of Physics, University of Sydney, Sydney, NSW 2006, Australia; svetlana.postnova@sydney.edu.au

**Keywords:** sleep, modelling, sleep debt, homeostatic pressure, napping, ageing, sleep propensity

## Abstract

Fixed sleep schedules with an 8 h time in bed (TIB) are used to ensure participants are well-rested before laboratory studies. However, such schedules may lead to cumulative excess wakefulness in young individuals. Effects on older individuals are unknown. We combine modelling and experimental data to quantify the effects of sleep debt on sleep propensity in healthy younger and older participants. A model of arousal dynamics was fitted to sleep data from 22 young (20–31 y.o.) and 26 older (61–82 y.o.) individuals (25 male) undertaking 10 short sleep–wake cycles during a 40 h napping protocol, following >1 week of fixed 8 h TIB schedules. Homeostatic sleep drive at the study start was varied systematically to identify best fits between observed and predicted sleep profiles for individuals and group averages. Daytime sleep duration was the same on the two days of the protocol within the groups but different between the groups (young: 3.14 ± 0.98 h vs. 3.06 ± 0.75 h, older: 2.60 ± 0.98 h vs. 2.37 ± 0.64 h). The model predicted an initial homeostatic drive of 11.2 ± 3.5% (young) and 10.1 ± 3.5% (older) above well-rested. Individual variability in first-day, but not second-day, sleep patterns was explained by the differences in the initial homeostatic drive for both age groups. Our study suggests that both younger and older participants arrive at the laboratory with cumulative sleep debt, despite 8 h TiB schedules, which dissipates after the first four sleep opportunities on the protocol. This has implications for protocol design and the interpretation of laboratory studies.

## 1. Introduction

The alternating 24 h pattern of sleep and wakefulness in humans has been attributed to the interplay between circadian and sleep homeostatic drives [1,2,3]. In the past four decades, the two-process model of sleep–wake regulation has served as the main conceptual framework to understand the effects of sleep deprivation, circadian phase, and/or inter-individual differences in sleep propensity profiles. In this model, a sleep homeostatic drive interacts with the circadian timing system to achieve consolidated periods of sleep during night-time and a continuous period of wakefulness (typically 16 h) during the daytime. Sleep homeostasis is expressed by a monotonic increase in sleep pressure during sustained wakefulness and its dissipation during (mainly slow-wave) sleep [4]. The circadian master clock provides temporal organization to the sleep–wake cycle through adaptive arousal mechanisms that oppose the homeostatic drive for sleep [3,5] by generating an intrinsic sleep–wake propensity rhythm that is entrained to 24 h, mainly by the light–dark cycle [6].

Perturbations in the circadian and homeostatic processes have been suggested to be linked to age-related changes in the sleep–wake cycle [7]. Ageing is associated with a decrease in nocturnal sleep duration and slow wave sleep and a decreased ability to sustain sleep [8,9]. Additionally, the circadian rhythm tends to advance with age, and its amplitude decreases [7].

Napping or short-cycle forced desynchrony protocols have been used to assess the circadian modulation of sleep propensity levels by keeping participants under near-constant homeostatic sleep pressure [10]. Prior to these protocols, participants are usually required to follow a regular sleep schedule to stabilise circadian timing, homogenize sleep pressure levels across participants, and thereby reduce individual variability and the probability of disproportionally accumulated sleep debt [11]. Participants’ adherence to these schedules is verified by actigraphy and sleep diaries during the 1–3 weeks preceding the laboratory visit. According to the two-process concept, these regular sleep schedules with an 8 h time in bed (TIB) allow the homeostatic drive to be sufficiently low at the start of the multiple nap protocol to achieve a consolidated wake period over the first protocol day and sleep is not induced until the following evening [1]. However, daytime sleep is generally observed during sleep opportunities on the first morning and afternoon of these protocols [12,13,14,15].

Mathematical models are valuable tools to help clarify concepts, challenge accepted ideas and aid in interpreting experimental and field data by extrapolating underlying regulation processes [16,17,18]. The two-process model provides insights into many aspects of sleep–wake regulation, including normal sleep–wake cycles, response to sleep deprivation, and the effect of naps on subsequent sleep [19]. Advances in the neurophysiological understanding of the sleep–wake switch led to the development of biophysical models that build on the two-process model and incorporate the interplay between mutual inhibitory sleep-promoting hypothalamic neurons and the ascending arousal system [20,21,22,23,24,25,26]. These models have been used to capture and predict sleep dynamics during normal sleep–wake cycles, shift work [27], spontaneous internal desynchrony [22], forced desynchrony (e.g., [24,26]), and sleep deprivation [28,29], as well as the impact of demographical characteristics such as age on these processes [30]. The model of arousal dynamics, in particular, accurately reproduces sleep propensity during forced desynchrony and multiple nap protocols in healthy, mainly young, adults [24]. However, even this model does not predict daytime sleep at the very start of the napping routine after an 8 h night-time sleep opportunity, which is expected to deplete the homeostatic drive. This observation challenges the existing assumptions about dynamics in homeostatic sleep pressure build-up and dissipation and associated parameter values used in bio-mathematical models. In parallel, while chronic sleep restriction studies indicate that an 8 h TIB may lead to cumulative excess wakefulness [31], the impact of higher-than-expected sleep debt at the start of the study on sleep propensity profiles over subsequent protocol days has not yet been assessed.

This study aimed to investigate how varying homeostatic sleep pressure at the start of a 40 h multiple napping protocol affects sleep propensity profiles over protocol days and to explore potential implications for the interpretation of experimental and field studies. We used the model of arousal dynamics to investigate whether the experimentally observed group average and individual sleep profiles could be reproduced by varying levels of homeostatic sleep pressure at the start of the protocol. We hypothesized that increasing homeostatic sleep pressure at the start of the study would decrease prediction error, especially for observed daytime sleep during the first protocol day. Considering that the wake-dependent homeostatic sleep pressure build-up and overall sleep capacity has been suggested to reduce with age [30], we applied the model to two datasets: one composed of a group of younger adults and the other composed of a group of older adults, both of whom underwent similar 40 h multiple nap protocols. It was expected that 8 h TIB schedules would induce higher than well-rested homeostatic sleep drive in the younger group, but not necessarily in the older group.

## 2. Results

### 2.1. Observed Total Sleep Time over the Baseline Night and Subsequent Nap Sleep Opportunities

**Figure 1:**
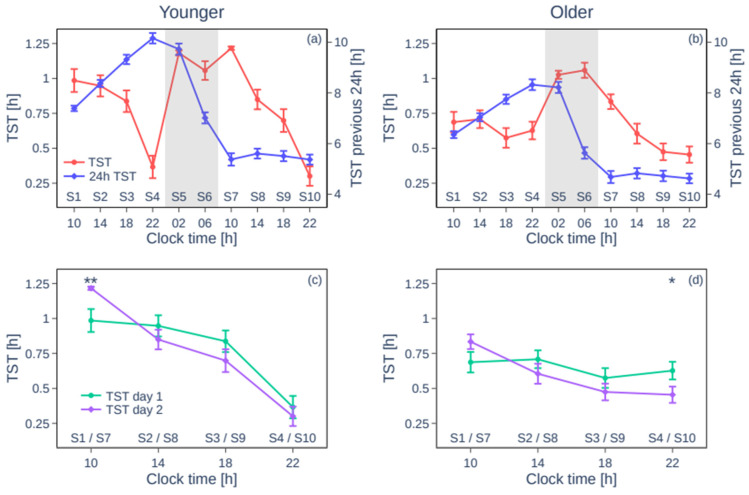
Sleep duration and sleep history over the multiple nap protocol. The top panels show the group mean total sleep time (TST) per sleep opportunity (red) and mean cumulative sleep time over the prior 24 h (blue) (±SEM) throughout the protocol. The bottom panels compare the TSTs during the sleep opportunities appearing at the same clock time on the first (S1–S4) and the second (S7–S10) day (with significant differences indicated with * for *p* < 0.05 and ** for *p* < 0.01). Panels (**a**,**c**) are for the younger group, and (**b**,**d**) are for the older group. Clock times are averaged protocol times.

As expected, TST (mean ± SD) during the baseline night was higher for the younger (7.39 ± 0.50 h) than for the older (6.35 ± 0.69 h) group (t(24.4) = −5.18, *p* < 0.001). Time courses of mean TST per nap sleep opportunity over the protocol are shown in Figure 1a (younger) and Figure 1b (older). In addition to a main effect of group (younger > older; F(1,45.7) = 8.91, *p* < 0.005), a main effect of nap session was observed (F(9,341) = 34.81, *p* < 0.0001, see also Table 1 for TST values). Overall, in both groups, the highest TST was observed during S5–S7, which are scheduled around the biological night. Finally, a group*session interaction (F(9,341) = 6.57, *p* < 0.001) indicated that the ability to sleep at different circadian times is influenced by age. While groups significantly differed during S1–S5 and S7–S9 (younger > older except for S4, all *p* < 0.05), no significant differences appeared for S5 (*p* = 0.08), S6 (*p* > 0.05) and S10 (*p* = 0.1). Finally, a day-by-day comparison by group revealed that the total amount of TST across S1–S4 (day 1) and S7–S10 (day 2) was not significantly different between days in both the younger (t(21) = 0.35, *p* = 0.73) and the older group (t(25) = 1.62, *p* = 0.12, see Table 1 and Figure 1c for the younger group and Figure 1d for the older group).

The similar daytime TST on day 1 and day 2 could result from similar prior sleep, and hence homeostatic drive, on these days. To investigate this, we compared accumulated 24 h TST on consecutive days (see Figure 1). For cumulative 24 h TST, a main effect of group (F(1,59) = 27.62, *p* < 0.0001) and of session (F(9,416) = 361.93, *p* < 0.0001), as well as a significant group*session interaction (F(9,416) = 6.83, *p* < 0.0001), was detected. For all sessions, accumulated 24 h TST was higher in younger than in older individuals. Higher accumulated TST was observed for all session-by-session comparisons between day 1 and day 2 (see Table 1). More specifically, for the younger group (Figure 1a), the 24 h TST increased over the first day from 7.39 ± 0.50 h before S1 (the baseline night) to 10.2 ± 1.0 h in the evening and then decreased during the biological night to 5.4 ± 1.1 h while staying at this level on the second day. The dynamics were similar for the older group, with the 24 h TST changing from 6.4 ± 0.7 h to 8.3 ± 1.1 h and 4.7 ± 1.2 h, respectively (Figure 1b). Thus, similar daytime sleep is observed during day 1 and day 2, despite higher 24 h TST at day 1, compared to day 2.

To assess the effect of circadian phase, we re-analysed the sleep profiles relative to DLMOn. The observed daytime sleep profiles on the first and second day of the protocol appear not to be due to differences in circadian phase between participants, as very similar TST dynamics are observed when the data are aligned to DLMOn time (Figure S1).

### 2.2. Modelling Varying Homeostatic Sleep Pressure at the Start of the Study: Impact on Daytime Sleep

The simulation of the protocol using the model of arousal dynamics with the default parameter values, as tuned to data from healthy young individuals, predicts no sleep in S1–S3 and TSTs comparable to the experimental data from S4 onwards [24]. Furthermore, no change in model parameters, e.g., circadian or homeostatic connection strength, allows for sleep to occur during S1–S3 (day 1), provided that the model demonstrates a stable nocturnal sleep pattern with a sleep duration of ~8 h outside of the protocol.

**Figure 2:**
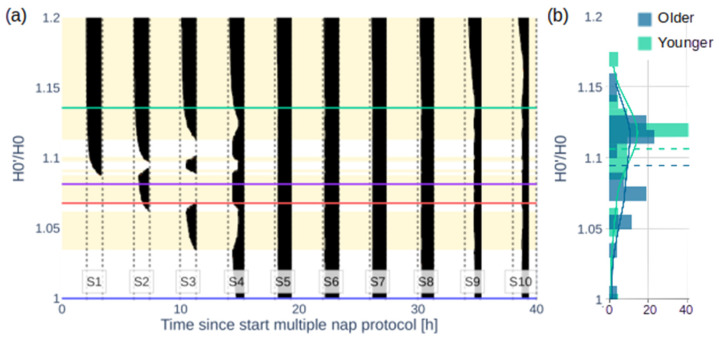
Effect of the initial homeostatic drive on sleep patterns on the multiple nap protocol. (**a**) Modelled sleep–wake pattern (with sleep in black and wake in white/yellow) over the 40 h multiple nap protocol as a function of the initial homeostatic drive H_0_′/H_0_. For H_0_′/H_0_ = 1, no sleep debt is introduced, and no sleep occurs in S1–S3 (blue line). Increasing H_0_′/H_0_ changes the sleep–wake pattern in S1–S4 and increases the TST over the protocol. The alternating yellow and white background colours indicate the areas with distinct sleep–wake patterns. The coloured lines correspond to the time courses of Figure 3. (**b**) Histogram of the best fit H_0_′/H_0_ values for the younger and older participants.

**Figure 3:**
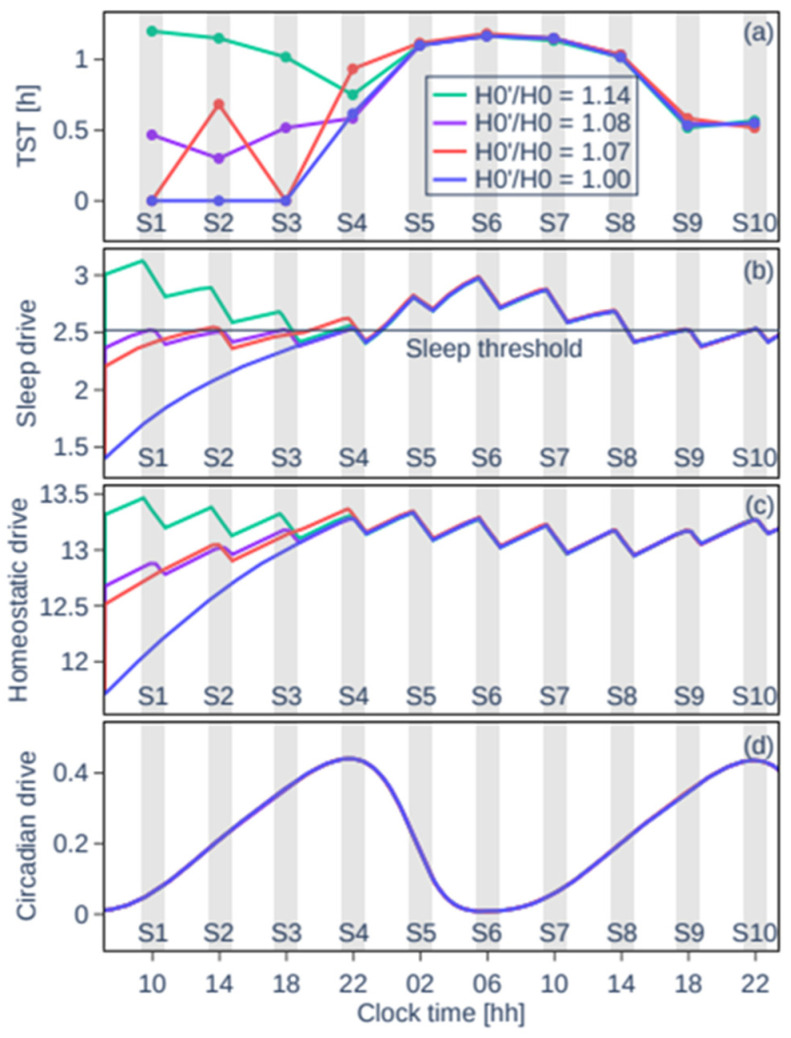
Time courses from the arousal dynamics model during the 40 h multiple nap protocol. For four different initial homeostatic drives: no sleep debt (blue), a low sleep debt (red), slightly higher sleep debt (purple), and a high sleep debt (green). (**a**) The TST shows four distinct modelled sleep behaviour patterns. (**b**) The sleep drive determines that sleep will occur when the sleep threshold is reached during a sleep opportunity. A slight variation in sleep drive can result in very different sleep profiles. The sleep drive consists of the homeostatic drive (**c**), the circadian drive (**d**), and a constant. (**c**) The homeostatic drive is modified to model the sleep debt at the start of the protocol. (**d**) The circadian drive is not affected by the sleep debt.

**Figure 4:**
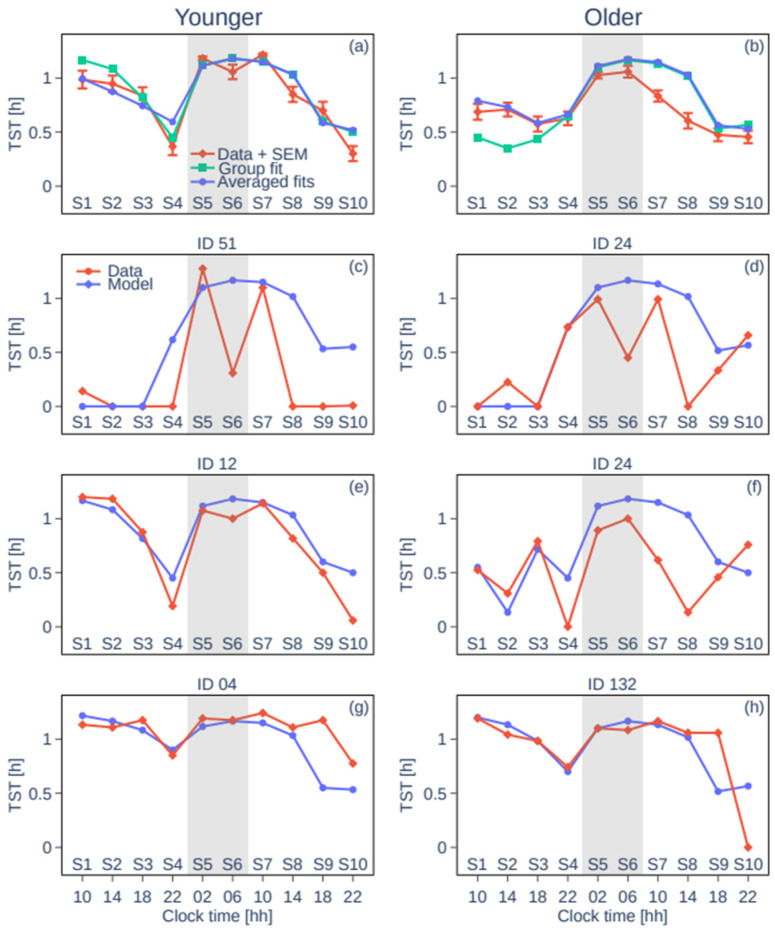
Total sleep times and model fits for group average data and representative individuals. Panels (**a**,**b**) show the group mean TST per sleep opportunity over the protocol with the SEM (red), the group mean fit (green), and the mean of the individual fits (blue). Left panels show data for younger participants and right panels show data for older participants. Panels (**c**,**d**) show participants with little sleep debt, panels (**e**,**f**) show participants with average sleep debt, and panels (**g**,**h**) show participants with large sleep debt.

As shown in Figure 2, Figure 3 and Figure 4, the experimentally observed TST profile can be accurately reproduced in the model by increasing the homeostatic drive at the start of the protocol, H_0_ (=11.72 mV), which simulates a pre-existing sleep debt. More precisely, increasing H_0_ to H_0_′ at the start of the multiple nap protocol allows for a variety of TST patterns to occur in S1–S4, like those observed in individuals (Figure 2a, histogram of the best-fit individual H_0_′/H_0_ in Figure 2b).

Introducing a sleep debt by increasing H_0_′, changes the predicted sleep–wake behaviour for S1–S4, demonstrating a systematic change in sleep patterns as shown in Figure 2a. The various H_0_′/H_0_ ranges lead to distinct sleep patterns:

- For 1 ≤ H_0_′/H_0_ < 1.034, sleep is predicted only starting from S4 onwards;
- For 1.034 ≤ H_0_′/H_0_ < 1.060, sleep appears and TST increases in S3 and decreases (but is non-zero) in S4;
- For 1.060 ≤ H_0_′/H_0_ < 1.065, sleep starts to appear in S2, which leads to decreasing TST in S3 to zero and increasing TST in S4;
- For 1.065 ≤ H_0_′/H_0_ < 1.079, TST increases in S2 while sleep is no longer generated in S3 and stays constant in S4;
- For 1.079 ≤ H_0_′/H_0_ < 1.081, sleep is initiated in S1 and decreases in S2;
- For 1.081 ≤ H_0_′/H_0_ < 1.084, sleep starts occurring in S3 and sleep increases in S1, while sleep decreases in S2 and S4;
- For 1.084 ≤ H_0_′/H_0_ < 1.090, sleep is no longer generated in S2;
- For 1.090 ≤ H_0_′/H_0_ < 1.096, TST increases in S1, S2, and S4 while TST decreases in S3;
- For 1.096 ≤ H_0_′/H_0_ < 1.109, sleep is no longer generated in S3;
- For 1.109 ≤ H_0_′/H_0_ < 1.201, sleep is initiated in S3 again, first decreasing but ultimately increasing TST in S4;
- For 1.201 ≤ H_0_′/H_0_ < 1.305, maximum sleep (>90% of the time) is achieved in S1–S4;
- For H_0_′/H_0_ ≥ 1.305, maximum sleep is achieved in all nap sleep opportunities S1–S10.

The patterns seen in Figure 2a are explained by the interplay of the model’s homeostatic and circadian drives with the sleep threshold, as shown in examples in Figure 3. The circadian drive is not affected by the initial homeostatic drive, Figure 3d. However, the total sleep drive, which is a weighted sum of the circadian and homeostatic drives plus a constant (for equations see Supplementary Materials), is affected as shown in Figure 3b. Without the sleep debt at H_0_′/H_0_ = 1, the homeostatic drive and the sleep drive build up until the sleep drive reaches the sleep threshold and sleep is initiated in S4 (blue curves). After S4, the circadian drive abruptly decreases at the start of the biological night, the sleep drive increases, and the TST per nap increases. This decreases the total sleep drive but it remains close to the threshold the following day due to the short sleep opportunities. With a high sleep debt, e.g., H_0_′/H_0_ = 1.14 (green curve in Figure 3b), the sleep drive starts well above the sleep threshold and sleep is initiated at every sleep opportunity until the end of the protocol.

For intermediate values, a small difference in H_0_′/H_0_ can result in very different sleep patterns on day 1 of the protocol when they are close to transitions as seen in Figure 2a and described above. For example, for H_0_′/H_0_ = 1.08, the sleep threshold is reached about halfway through S1, dissipating only a small part of the homeostatic pressure such that for S2 the sleep threshold is reached again and the process repeats and sleep is initiated in every sleep opportunity (purple curve in Figure 3b). While for H_0_′/H_0_ = 1.07, there is no sleep in S1, which results in a higher sleep pressure at the start of S2 and a large amount of sleep decreasing the sleep pressure for S3 (red curve in Figure 3b).

Fitting H_0_′ in the model to the experimental data allows us to reproduce both the group average and individual TST patterns for the younger and the older groups in S1–S4 (Figure 4). Group average profiles and model fits are shown in Figure 4a,b. The lowest RMSE is found for H_0_′/H_0_ = 1.123 and 1.082 (RMSE of 0.24 and 0.45) for the younger and older groups, respectively. Individual sleep patterns match those predicted by the model with varying values of H_0_′/H_0_. The distribution of H_0_′/H_0_ for the best individual fits is shown in Figure 2b and is aligned with the respective sleep patterns in Figure 2a. The mean H_0_′/H_0_ is 1.112 ± 0.035 with an RMSE of 0.39 ± 0.24 for the younger group and 1.101 ± 0.035 with an RMSE of 0.38 ± 0.16 for the older group. Examples of the individual sleep patterns and model fits are shown in Figure 4c–h; all individual fits are shown in Figures S2 and S3. The average across the individual fits produces an even better agreement with the experimental group average data, with the RMSE decreasing to 0.26 and 0.11 for the younger and the older groups, respectively (Figure 4a,b).

### 2.3. Wash-Out Effect of Modelled Sleep Debt in the Multiple Nap Protocol

To improve our understanding of the effects of increasing sleep debt over time on the protocol, we compared the modelled time courses of the homeostatic drive with and without sleep debt. Figure 2 and Figure 3 show that the difference in TST diminishes over the protocol time and seems to disappear by S5, indicating that higher-than-expected sleep debt is washed out during S1–4.

**Figure 5:**
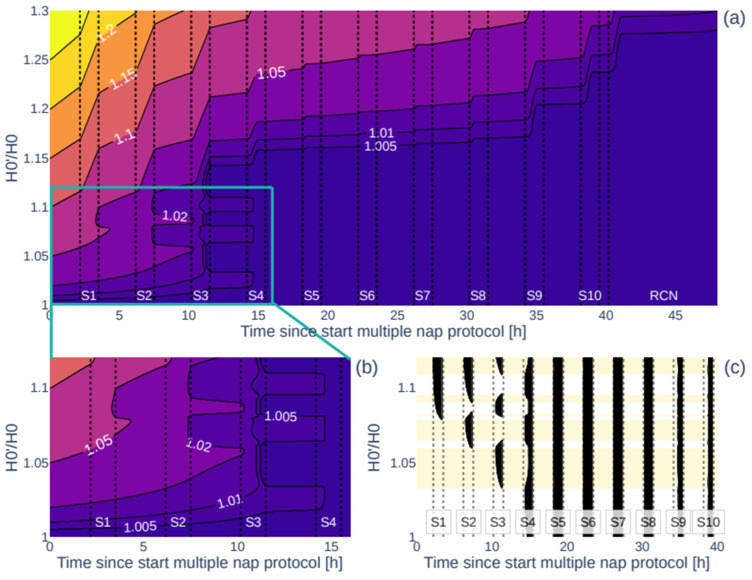
Wash-out effect of sleep debt during the multiple nap protocol. (**a**) A contour map demonstrating the effects of the initial homeostatic drive H_0_′/H_0_ and time into protocol on the dynamics of H’/H—the homeostatic variable H’ relative to its default value, H. Darker colour indicates a smaller H’/H value with H’/H = 1 indicating that H’ converged to H and the effect of the initial sleep debt is no longer present. (**b**) Zoomed in section of the contour map in (**a**). (**c**) Corresponding sleep–wake pattern. The alternating white and yellow background colours indicate the different modes as separated by the sleep thresholds.

To investigate how such wash-out depends on the initial sleep debt, Figure 5a shows how H’/H, the ratio of the homeostatic drive with an initial sleep debt over the default drive without sleep debt, changes over the protocol time depending on the initial ratio of H_0_′/H_0_. In general, the larger H_0_′/H_0_ at the start of the protocol, the more sleep debt at the start, and the longer it takes for the sleep debt to dissipate and for H’ to return to the default H. For H_0_′/H_0_ < 1.16, H’/H drops to below 1.01 (less than 1 percent) starting from S4, meaning that enough sleep is achieved within the first four sleep opportunities to dissipate the additional sleep debt. For higher H_0_′/H_0_, sleep in S1–S4 is insufficient to dissipate the extra sleep debt, and S5–S8 appear during the biological night when TST is already maximal for both groups. For these values of H_0_′/H_0_, the sleep debt wash-out occurs at S8 and later.

For H_0_′/H_0_ < 1.12, the wash-out period varies between three and four sleep opportunities, depending on the sleep pattern (Figure 5b,c). Sleep dissipates homeostatic pressure, and the timing of sleep changes the course of the homeostatic pressure, which affects the timing of the wash-out. Sleep earlier in the protocol results in an earlier decrease in the homeostatic pressure, which causes the sleep thresholds to align with the irregularities in the boundaries.

## 3. Discussion

By using a model-based approach, we assessed the impact of varying sleep pressure levels on daytime sleep propensity profiles. We focused on sleep patterns over the first day in a multiple nap protocol, which could not be explained by the two-process concept in its default state. The effects of sleep debt on recovery sleep have been modelled previously with conceptual and physiologically based models [2,29,32]. These models correctly predict the effects of acute sleep deprivation on recovery sleep duration in groups of long, regular, and short sleepers [32]; however, the effect of sleep debt on short-cycle forced desynchrony protocols has not been investigated. The two-process concept of sleep regulation assumes the homeostatic drive to be sufficiently low at the start of the multiple nap protocol to not induce sleep until the following evening [1]. Similarly, the arousal dynamics model, when entrained to a regular diurnal cycle, is not able to predict daytime sleep as we observe in the protocol without changing the homeostatic pressure (and/or without assuming higher than expected sleep debt at study start).

Tuning physiology-derived model parameters has been shown to account for different sleep timing [33], and modifications to circadian and homeostatic parameters can explain changing sleep patterns related to ageing [30]. Polyphasic mammalian sleep can be modelled by an arousal dynamics model [34], and the two-process model of sleep–wake regulation can simulate a range of different sleep–wake cycles, including cycles with multiple sleep episodes each day [1]. However, these modifications would not reproduce normal human diurnal cycles in unconstrained settings. Our results show that sleep debt at the start of the study can explain sleep propensity profiles during the first protocol day.

By varying H_0_, we directly manipulated the homeostatic pressure at the start of the protocol, which can originate from acute sleep debt caused, for example, by insufficient sleep during a baseline night in the laboratory or from sleep debt induced by previous chronic sleep restriction. Our results point to the existence of slowly accumulating sleep debt, rather than to the effects of acute sleep deprivation induced by the baseline night. The accumulated TST over the previous 24 h, a metric representing recent sleep history and inversely related to recent sleep debt, increases for both groups past the expected TST for a normal night on the first protocol day [35]. Thus, similar daytime sleep patterns are observed on day 1 and day 2 of napping protocols despite a much shorter prior 24 h sleep history on day 2 compared to day 1. In other words, the cumulative TST decreases towards the second protocol day without a concomitant increase in daytime sleep. This indicates accumulated sleep debt before the start of the protocol, rather than the sole effect of acute sleep debt induced by a night of sleep in laboratory settings.

This accumulated sleep debt is predicted to increase the H_0_′/H_0_ ratio to 1.112 ± 0.035 (younger) and 1.101 ± 0.035 (older). As seen in Figure 2 and Figure 3, these values are similar to those observed at the start of day 2, which explains the similar daytime sleep duration. However, the mechanisms behind the increased homeostatic drive at the start of each day are different. On day 1, the sleep debt is likely caused by the prior 8 h fixed TIB schedules, which are known to increase cumulative sleep debt [31]. This sleep debt dissipates after the first four naps, as shown in Figure 4. On day 2, the elevated values of the homeostatic drive are caused by the short sleep–wake cycles that reduce the variation in the homeostatic drive, but increase its mean daily values as seen in Figure 3.

This explanation is consistent with the observed higher variance in daytime sleep duration on day 1 compared to day 2: 3.14 ± 0.98 h (SD is 31% of the mean) vs. 3.06 ± 0.75 h (25%) in the younger group, and 2.60 ± 0.98 h (38%) vs. 2.37 ± 0.64 h (27%) in the older group. On day 1, the individual variability is caused by both the physiological differences and the differences in prior sleep debt, while on day 2, the sleep debt is more homogenous due to the napping protocol, resulting in reduced variance. We thus propose that the day 2 sleep propensity profile is more representative of underlying physiological mechanisms.

The observed H_0_′/H_0_ values are below the homeostatic pressure reached after a single night of total sleep deprivation, which, according to model simulations, would result in H_0_′/H_0_ = 1.179. Assuming a linear increase in H during sleep deprivation and a saturated sleep duration of 8.5 h in the model [31,36,37], the observed value of H_0_′/H_0_ = 1.1 would correspond to extending wake time by 4.76 h. According to Van Dongen et al. [31], such an excess of wake time could be achieved after 10 days of 8 h sleep per night, or just 3 days of 7 h of sleep in the young. For the older group, 7.2 h a night is expected [35] and 4.76 h of excess wake could be achieved by 7 days of 6.5 h of sleep per night. The fixed sleep schedule of an 8 h time in bed per night is thus not expected to introduce the observed additional sleep pressure in the older group. However, only 6.3 h of sleep is observed during the baseline night in the older group. Ageing is suggested to affect the ability to sustain sleep over prolonged periods and to increase sleep fragmentation [38], which could result in higher sleep debt in the older group despite sufficient sleep opportunity.

The observed sleep duration was significantly lower in the older group, compared to the younger group, during the baseline night, but also during the protocol days (except for S4, scheduled during the wake maintenance zone). Thus, as expected, under equal conditions, older individuals produce less sleep over sleep opportunities, thereby confirming the previous observations of overall reduced sleep capacity (e.g., [39]).

For individual predictions, the largest RMSEs in younger individuals were seen in participants with atypical behaviour, such as a very low TST overall or alternating between very high and very low TST during consequent naps. This could be explained by larger differences between these individuals and the default ‘standard individual’ parameters of the model. For the older participants, averaging individual fits (compared to model predictions based on group averages) led to an improvement in prediction errors (RMSE from 0.45 to 0.11 for the older group vs. 0.24 to 0.26 for the younger group). The increased inter-individual variability in sleep profiles reflects a hallmark of the ageing process [40] and may speak in favour of different compensatory mechanisms working to achieve sufficient sleep despite altered physiology. For example, while some older adults need less sleep, others may be unable to achieve the sleep that they need. Alternatively, other mechanisms, such as an altered circadian wake drive may be involved. Thus, potential age-related changes in sleep physiology and inter-individual variability should be taken into account in future work. For example, when considering all sleep opportunities, the prediction consistently overestimates sleep on day 2 in S7–S9 in the older group. This is because the default model parameters were calibrated for young adults and used here without further adjustment.

Another cause for discrepancies between the model predictions and the experiment could be the incompleteness of the model. It is possible that sleep could always be initiated in optimal conditions, e.g., when in bed in a dark room with a lack of stimuli and a relaxed state of mind, an increased homeostatic pressure might not be required to initiate sleep. Ambient temperature [41], body position, and available stimuli affect sleep propensity and arousal [42] and are not yet included in sleep models. However, the wake effort model parameter, as tuned for the correct protocol type [24], could partly account for these differences. Lastly, the mental state of participants during the protocol could play a role and participants could initiate sleep for pleasure or as a time filler [43].

A potential limitation of this study is that directly increasing H_0_ at the start of the protocol might result in a model state that cannot be reached by actual sleep behaviour. The variable H represents, together with other state variables, the state of the model, and directly adjusting this value might cause the model to jump to an uninterpretable state. However, decreasing the length of the baseline night and indirectly increasing H_0_ by the resulting sleep restriction leads to comparable results in sleep behaviour.

Overall, tuning the initial homeostatic pressure allowed for accurate predictions of the observed daytime sleep patterns in individuals and group averages. This emphasised the importance of adapting the duration of the sleep opportunities to group average or even individual needs, as sleep is affected by chronotype and other demographic factors such as sex and age. In practice, an additional 30 min on both sides of an individual’s habitual time in bed should provide enough sleep opportunity to prevent excess sleep restriction. However, sleep quality cannot be controlled for and participants could thereby still accumulate sleep debt, despite an appropriate sleep opportunity. This might also, at least partially, explain the observed sleep debt for the older adults. Incorporating extensive individual sleep history and investigating the correspondence between sleep debt and modelled homeostatic pressure could improve the understanding of inter-individual differences. Our results thereby have implications for the interpretation of experiments and may challenge existing theories about sleep homeostatic dynamics and its impact on sleep initiation and/or maintenance over the daytime.

## 4. Methods

### 4.1. Participants

Twenty-two younger (20–31 years; 10 male) and twenty-six older individuals (61–82 years; 15 men) underwent a 40 h multiple nap protocol at two different research centres (the younger participants at the Centre for Chronobiology of the University of Basel, and the older participants at the GIGA-CRC in Vivo Imaging of the University of Liège). All participants were free from current or past neurological, psychiatric, and sleep disorders, were non-smokers, and did not use regular medication or drugs, except for oral contraceptives and stable hypertension and/or hypothyroidism treatment for over 6 months. Participants did not engage in shift work or trans-meridian travel for 3 months prior to participation and were not habitual nappers. None of the younger participants were extreme chronotypes, while in the older group, 6 individuals were definite morning types. A screening night with polysomnography was used to screen for sleep disorders (Apnea–Hypopnea Index [AHI] < 15/h and Periodic Limb Movement [PLMS] index < 15/h) and was used as a habituation night. Young women were tested during the luteal phase of their menstrual cycle when not using oral contraceptives. The participants provided written informed consent and received financial compensation, and the study and procedures were approved by the local ethics committees and conformed with the Declaration of Helsinki.

### 4.2. Experimental Protocol

The detailed protocols have been published elsewhere (e.g., [13,14,44]). Participants were instructed to abstain from alcohol and caffeine for one week before entering the laboratory to prevent withdrawal effects, and to keep a fixed sleep–wake schedule based on their habitual sleep times (8 h of sleep opportunity with a maximal deviation of ±30 min) for at least 7 days to ensure sufficient sleep and stable circadian entrainment. Compliance with the schedule during the week preceding the laboratory visit was verified by actigraphic recordings (MotionWatch 8 for data acquired at ULiège, Actiwatch 7 for data acquired at UBasel). Sleep schedules in the laboratory (i.e., sleep and wake-up times for the baseline night and subsequent nap timing) were adapted such that the 8 h baseline night aligned with the individuals’ recorded night-time rest. After the baseline night, participants underwent a 40 h multiple nap protocol encompassing ten sleep–wake cycles of 80 min of sleep opportunity (i.e., a nap) alternating with 160 min of wakefulness. The first sleep opportunity started on average 130 min after the scheduled wake-up time from the baseline night. The duration of wakefulness in the last cycle was restricted to 40 min such that the recovery night started at a habitual sleep time.

The experiment was conducted under controlled laboratory conditions with respect to light exposure (dim light: <8 lux during scheduled wakefulness and 0 lux during scheduled sleep opportunities), the absence of external time cues, isocaloric food intake (standardized meals every 4 h), and body posture (semi-recumbent position during scheduled wakefulness and recumbent during naps). Participants were not allowed to stand up, except for regularly scheduled bathroom visits, and social interaction was restricted to communications with study personnel.

### 4.3. Melatonin

Melatonin was assessed hourly during scheduled wakefulness via saliva samples that were analysed for melatonin levels as described in previous publications (Ref. [44] for the younger group, and Ref. [45] for the older group). The timing of dim light melatonin onset (DLMOn) was defined as the point in time at which melatonin levels reached 50% of the fitted peak-to-baseline amplitude of an individual’s full-night melatonin profile [46].

### 4.4. EEG Data Acquisition and Analysis

During the multiple nap protocol, sleep was measured using 7 electroencephalographic (EEG) channels (Fz, C3, Cz, C4, Pz, Oz, and O2), as well as two bipolar electro-oculograms, and two bipolar submental electromyograms. Signals were recorded using V-Amp digital sleep recorders (Brain Products GmbH, Germany) with sintered MRI-compatible Ag/AgCl ring electrodes with a 15 kΩ resistor (EasyCap GmbH, Germany), and N7000 amplifiers (EMBLA, Natus Medical Incorporated, Planegg, Germany) with classical Ag/AgCl ring electrodes in Basel and Liège, respectively. The sampling rate was set at 500 Hz and signals were filtered by applying a 50 Hz notch filter. Sleep stages were automatically scored in 30 s epochs according to the American Academy of Sleep Medicine criteria (AASM [47]) using the ASEEGA sleep scoring algorithm (ASEEGA, PHYSIP, Paris, France). The latter has been previously tested against visual scoring for night-time sleep in younger [48,49] and older individuals [50], and more recently, also in the context of nap sleep [51].

The total sleep time (TST, the sum of sleep stages N1, N2, N3, and REM sleep) was extracted over the sleep opportunities (baseline night and 10 nap opportunities). To quantify recent sleep history and estimate potentially accumulated sleep debt during the protocol, prior 24 h TST was calculated for each sleep opportunity as the sum of TSTs in the 24 h window before the start of that sleep opportunity. For the first protocol nap (S1), only the baseline night was considered.

Finally, in addition to the analysis against time since waking, we re-analysed the sleep profiles relative to DLMOn time to assess the effect of circadian phase. The TST per sleep opportunity was calculated for the participants relative to their circadian phase, by extracting the TST in 4 h bins relative to the participants’ DLMOn. The prior 24 h TST for each of these bins was also extracted.

### 4.5. Statistical Analysis

Statistical analyses were performed with SAS 9.1 (SAS Institute, Cary, NC, USA). We used a general linear mixed model with PROC MIXED to determine the effects of group, session, and the group × session interactions, as fixed model effects, on TST and prior 24 h TST, with the subject effect as a random factor and session as a repeated effect and a first-order autoregressive variance–covariance matrix being specified. Differences of least square means were used to determine significant differences among sessions for both groups separately and at *p* < 0.05. More specifically, TST was compared between day 1 and day 2 of the multiple nap protocol, as the total sleep during nap sleep opportunities 1–4 (S1–S4; day 1) and 7–10 (S7–S10; day2).

### 4.6. Biomathematical Model

The model of arousal dynamics [24] was used to predict sleep–wake patterns over the protocol. The model simulates the sleep–wake switch by including the mutually inhibitory wake-active monoaminergic neurons (MA) and the sleep-active ventrolateral preoptic nucleus of the hypothalamus [52]. The activity of these neuronal populations is regulated by the homeostatic and the circadian drive, which combine into the sleep drive. Sleep is predicted when the combined sleep drive rises above a critical threshold. The model equations and parameters have been published in full elsewhere [24,53].

The model with a default parameter configuration predicts a regular diurnal sleep–wake cycle with ~8.5 h sleep starting at 23:30. The default light schedule is 250 lux from 8:00 to 20:00 and 40 lux otherwise while awake, and light is set to 0 lux during sleep [24,53]. To use the model for our multiple nap protocol, the wake effort parameter was set to 0.33 mV as optimized for short sleep–wake cycle protocols [24]. This parameter represents the minimum voltage of the wake-active MA during forced wake and does not affect model behaviour outside forced wakefulness.

At the start of the simulations, the model was in a rested and entrained state, demonstrating regular sleep–wake cycles. The protocol started on day 10 of the simulations, incorporating an 8 h baseline sleep opportunity centred on the model’s habitual sleep time, followed by 10 napping cycles as in the experiment. Wakefulness was forced during the scheduled 160 min wake episodes, and sleep was allowed during the 80 min sleep opportunities. The light level during the 40 h protocol was set to 3 lux.

To test the hypothesis that an increased sleep debt causes observed daytime sleep on day 1 of the multiple nap protocol, we varied the initial value of the homeostatic drive, H, at the start of the 40 h multiple nap protocol to a different value, H_0_′. The best-fit value was extracted by fitting the model-predicted sleep to the experimental data for the group average and individuals’ TST. The fit was achieved by minimizing the root-mean-squared error (RMSE) for S1–S4. The error calculation focused on day 1 daytime sleep, i.e., S1–S4, as these sleep opportunities could not be accurately predicted in previous reports. We calculated the best fit and corresponding RMSE for each individual and each group’s average TST per nap.

## Figures and Tables

**Table 1:** Total sleep time (TST) and accumulated 24 h TST in hours: comparisons across protocol days, per age group.

Older			Younger			
*p*-Value	Day 2, TST ± SD [h]	Day 1, TST± SD [h]	*p*-Value	Day 2, TST ± SD [h]	Day 1, TST± SD [h]	TST: Day 1 vs. Day 2
0.06	0.83 ± 0.27	0.69 ± 0.37	0.0070	1.22 ± 0.06	0.99 ± 0.39	S1 vs. S7 (10:00)
0.19	0.60 ± 0.36	0.71 ± 0.32	0.25	0.85 ± 0.33	0.95 ± 0.36	S2 vs. S8 (14:00)
0.20	0.47 ± 0.30	0.57 ± 0.36	0.11	0.70 ± 0.38	0.84 ± 0.36	S3 vs. S9 (18:00)
0.029	0.45 ± 0.30	0.63 ± 0.32	0.44	0.30 ± 0.33	0.37 ± 0.38	S4 vs. S10 (22:00)
0.12	2.37 ± 0.64	2.60 ± 0.98	0.73	3.06 ± 0.75	3.14 ± 0.98	S1–4 vs. S7–10 (Total)
*p*-Value	Day 2, 24 h TST± SD [h]	Day 1, 24 h TST± SD [h]	*p*-Value	Day 2, 24 h TST± SD [h]	Day 1, 24 h TST± SD [h]	24 h TST: Day 1 vs. Day 2
<0.001	4.68 ± 1.21	6.35 ± 0.69	<0.001	5.37 ± 1.14	7.39 ± 0.50	S1 vs. S7 (10:00)
<0.001	4.83 ± 1.05	7.04 ± 0.80	<0.001	5.60 ± 0.91	8.37 ± 0.65	S2 vs. S8 (14:00)
<0.001	4.72 ± 1.06	7.75 ± 0.94	<0.001	5.51 ± 0.95	9.32 ± 0.87	S3 vs. S9 (18:00)
<0.001	4.62 ± 0.97	8.32 ± 1.08	<0.001	5.37 ± 0.93	10.16 ± 0.97	S4 vs. S10 (22:00)

## Data Availability

The data underlying this article will be shared on request to the corresponding author.

## Supplementary Materials

The following supporting information can be downloaded at https://www.mdpi.com/article/10.3390/clockssleep7010002/s1, Figure S1: Sleep duration and sleep history over the multiple nap protocol relative to melatonin onset. Left hand panels show the group mean total sleep time (TST) per sleep opportunity and mean cumulative sleep time over the prior 24 h (±SEM) throughout the protocol. The right hand panels compare the TSTs during the sleep opportunities appearing at the same circadian phase on the first (S1–S4) and the second (S7–S10) day (with significant differences indicated with * for *p* < 0.05 and ** for *p* < 0.01). Panels (a) and (b) are for the younger group, and (c) and (d) are for the older group. Figure S2: Data (red) and best fits (blue) for younger participants. Participants are ordered by the best fit initial value of the homeostatic drive, H_0_. The root-mean-squared error over the first four sleep opportunities is provided. Figure S3: Data (red) and best fits (blue) for older participants. Participants are ordered by the best fit initial value of the homeostatic drive, H_0_. The root-mean-squared error over the first four sleep opportunities is provided.
