# Supplementary material for: Impact of Varying Sleep Pressure on Daytime Sleep Propensity in Healthy Young and Older Adults
Source: Clocks Sleep. 2025 Jan 2;7(1):2. doi: 10.3390/clockssleep7010002 (PMC11755553; doi:10.3390/clockssleep7010002)
Supplement: Supplementary file 1 [file clockssleep-07-00002-s001.zip › Supplementary_Material.pdf]

Supplementary material

Figure S1: Sleep duration and sleep history over the multiple nap protocol relative to melatonin onset. Left hand panels show the group mean total sleep time (TST) per sleep opportunity and mean cumulative sleep time over the prior 24 hours (±SEM) over the protocol. The right hand panels compare the TSTs during the sleep opportunities appearing at the same circadian phase on the first (S1-S4) and the second (S7-S10) day (with significant differences indicated with * for $p<0.05$, ** for $p<0.01$). Panels (a) and (b) are for the young group, and (c),(d) for the older.

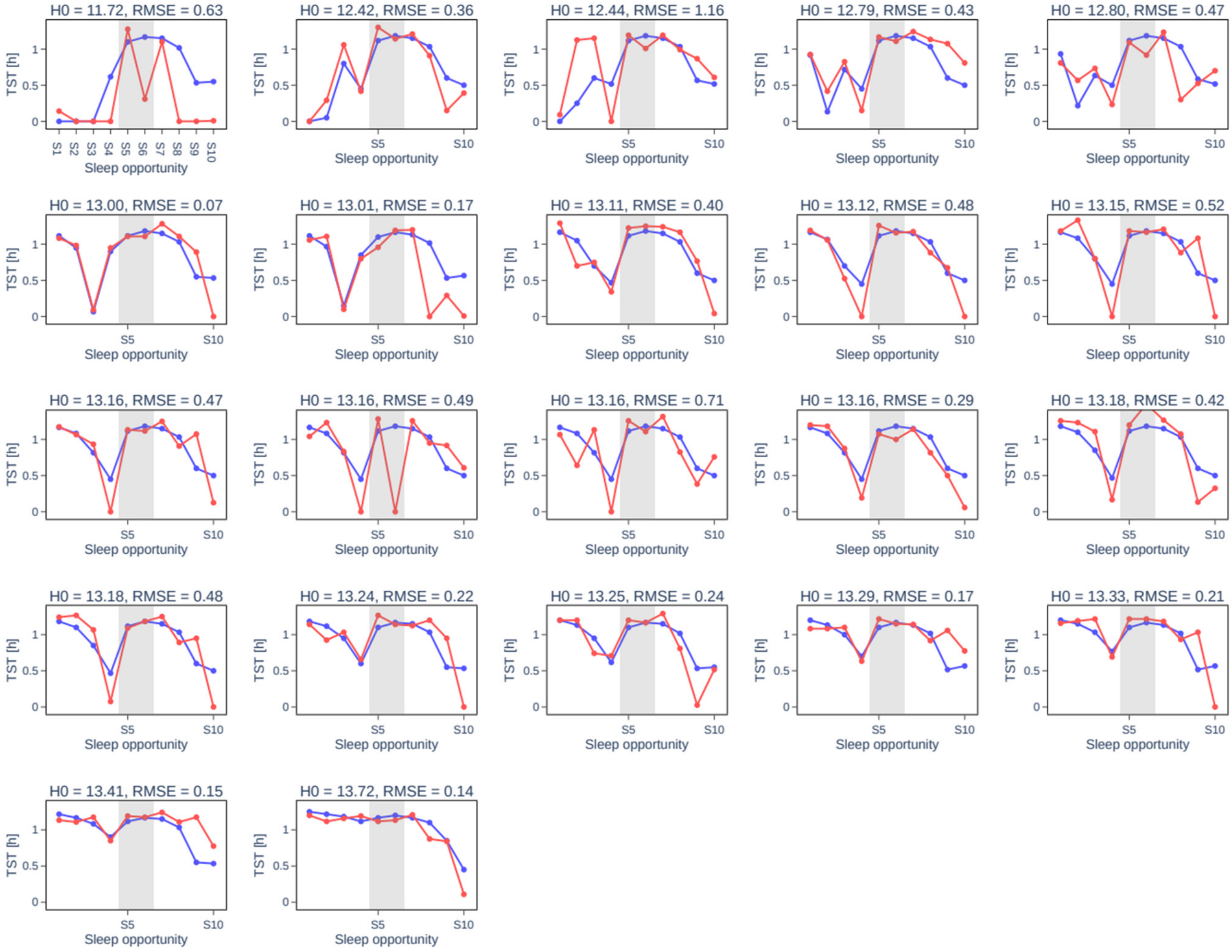

Figure S2: Data (red) and best fits (blue) for young participants. Participants are ordered by the best fit initial value of the homeostatic drive, H0. The root-mean squared error over the first four sleep opportunities is provided.

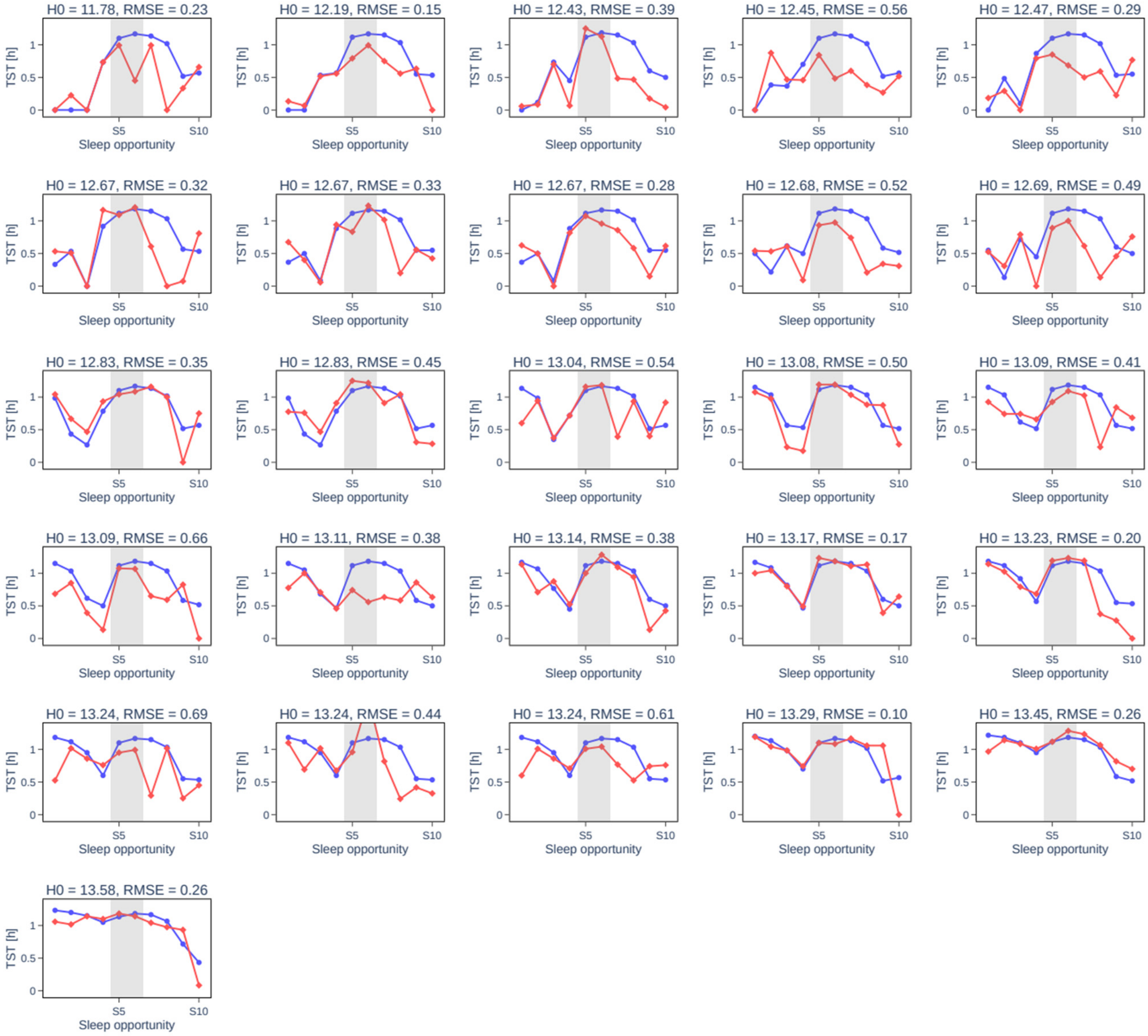

Figure S3: Data (red) and best fits (blue) for older participants. Participants are ordered by the best fit initial value of the homeostatic drive, H0. The root-mean squared error over the first four sleep opportunities is provided.
