# Supplementary figures and images for: Impact of Varying Sleep Pressure on Daytime Sleep Propensity in Healthy Young and Older Adults
Source: Clocks Sleep. 2025 Jan 2;7(1):2. doi: 10.3390/clockssleep7010002 (PMC11755553; doi:10.3390/clockssleep7010002)

# Young

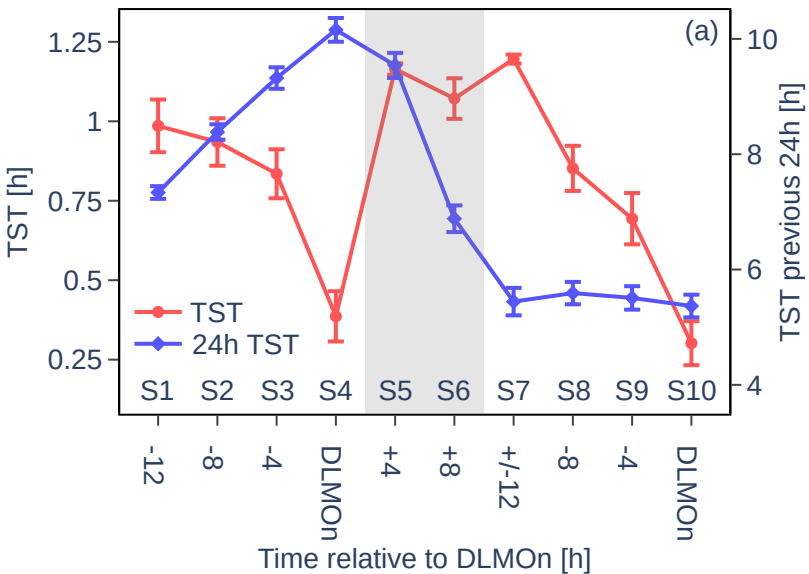

# Older

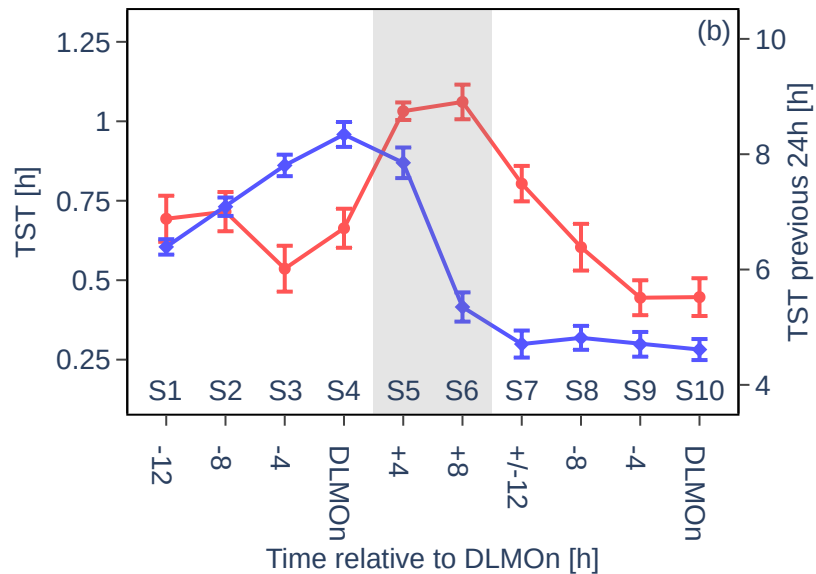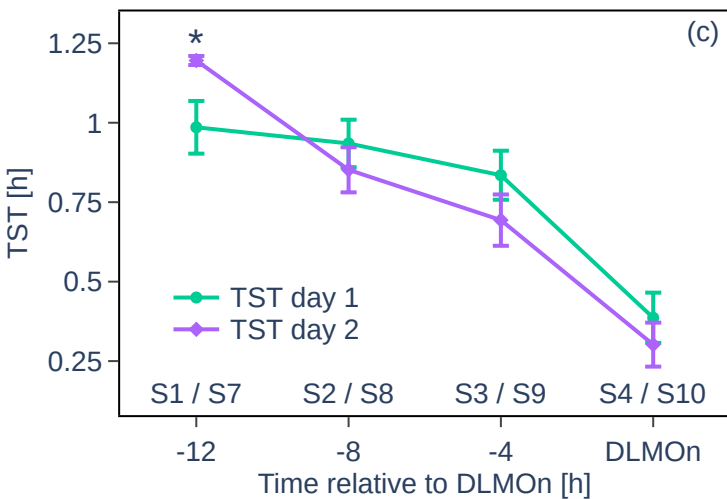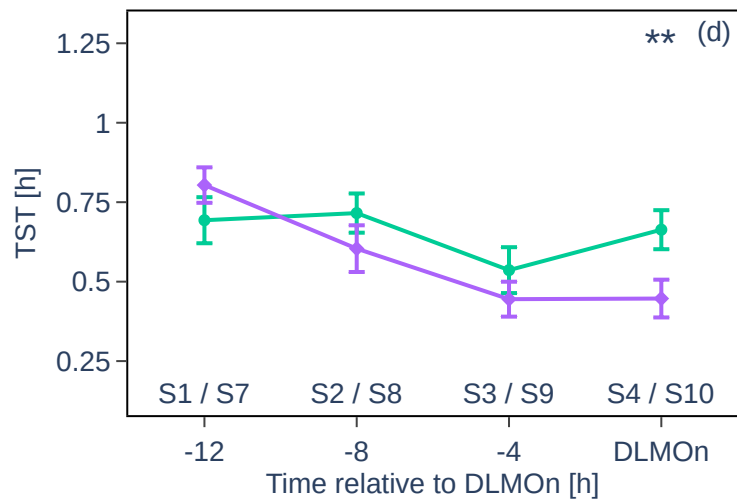

Supplement: Supplementary file 1 [file clockssleep-07-00002-s001.zip › FigureS1_SleepOverProtocol_DLMOn.pdf]

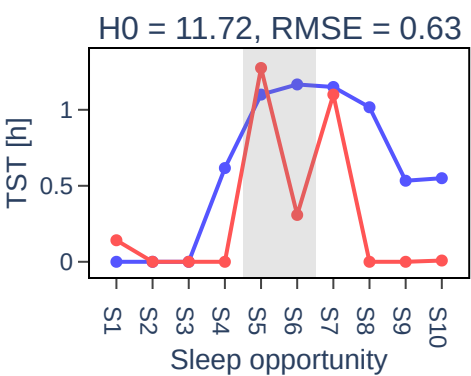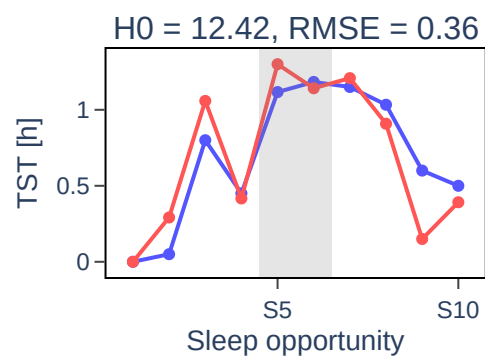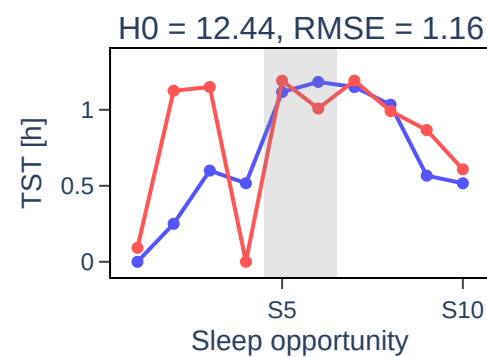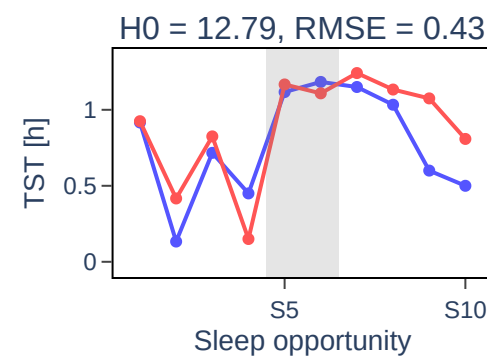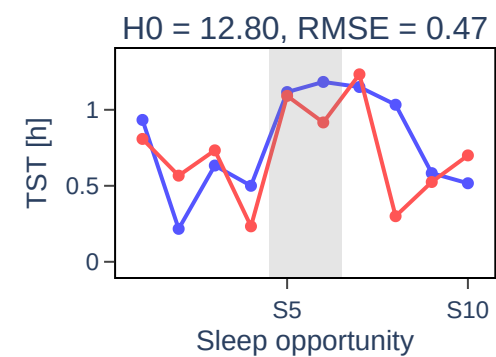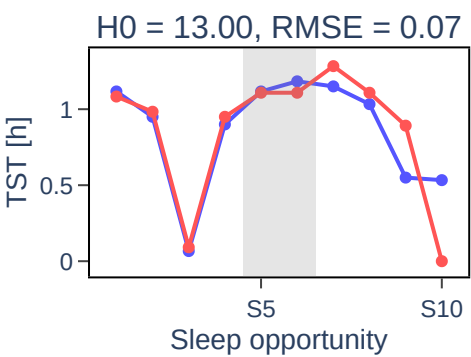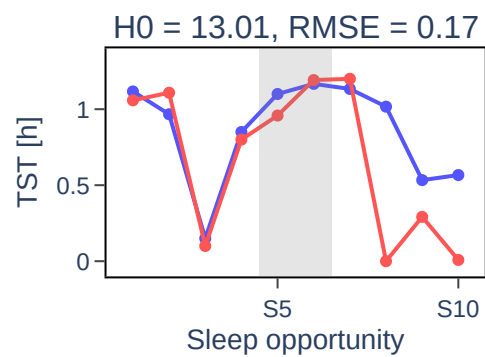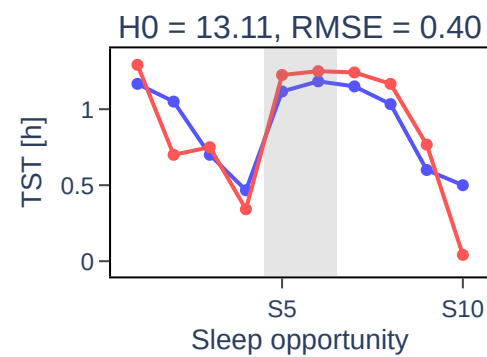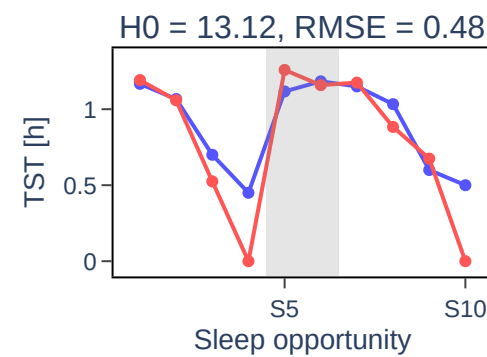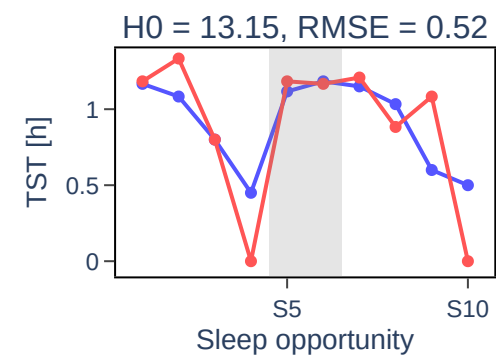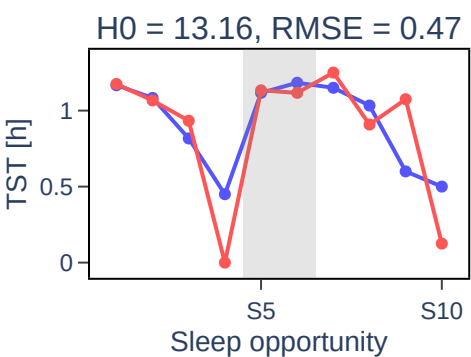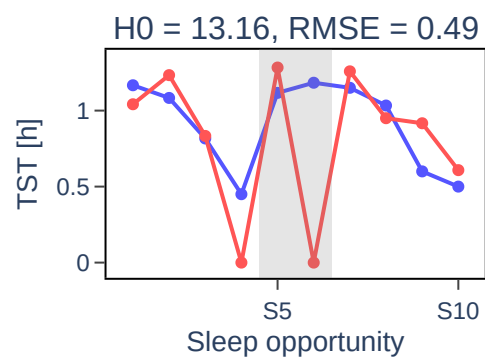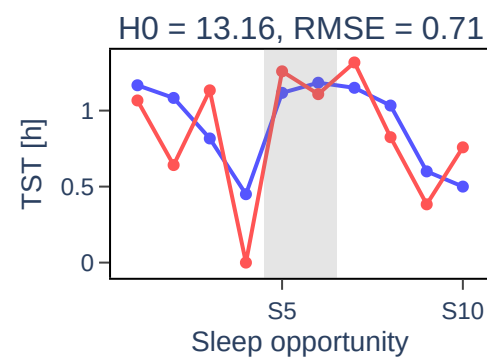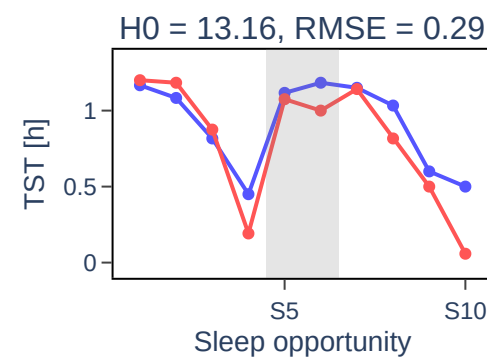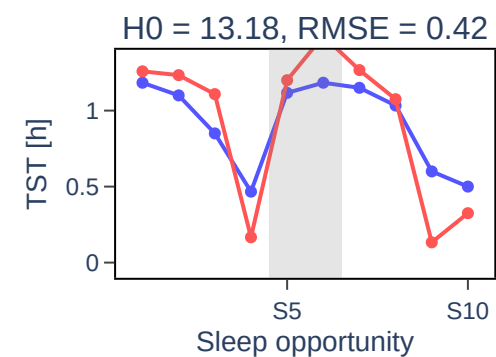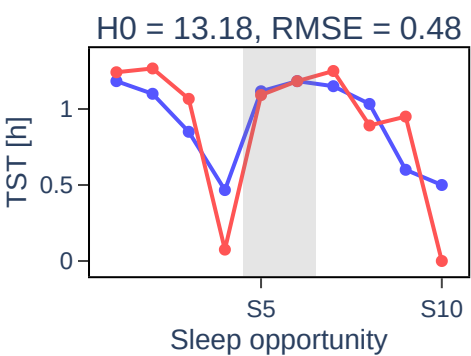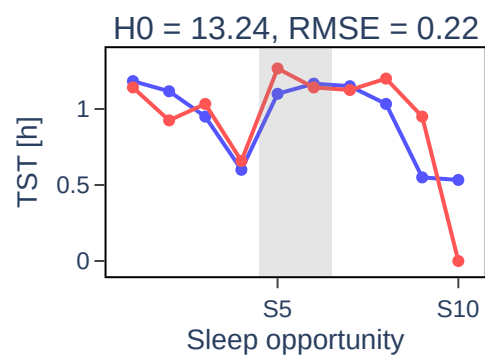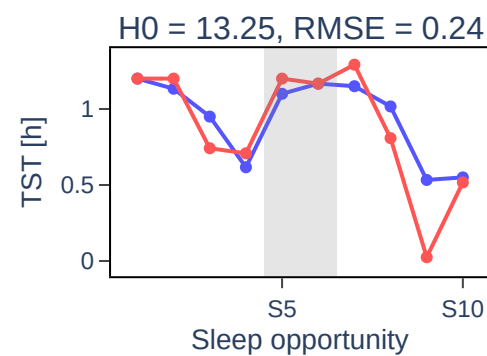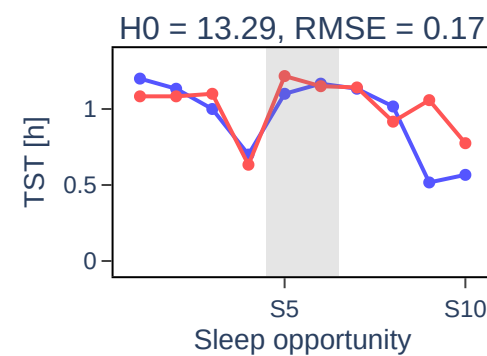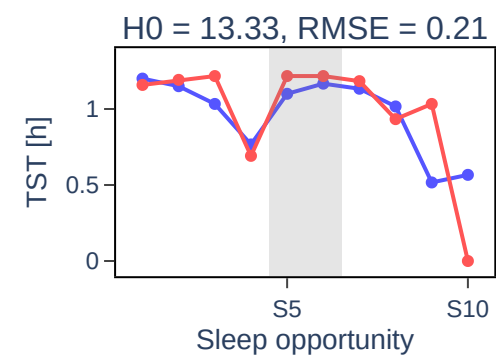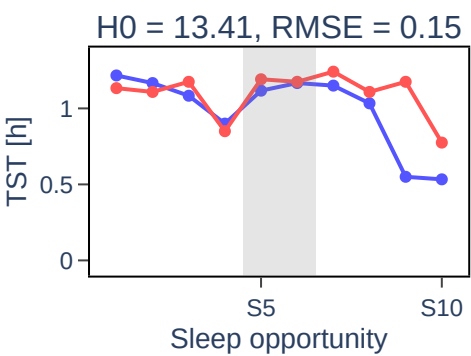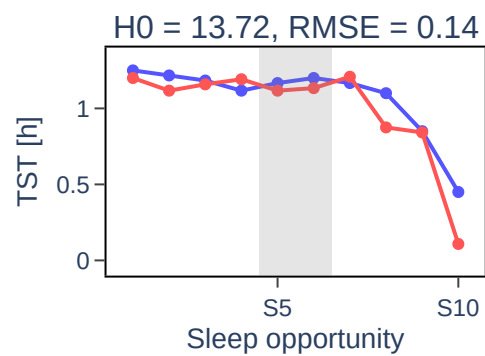

Supplement: Supplementary file 1 [file clockssleep-07-00002-s001.zip › FigureS2_YoungParticipants.pdf]

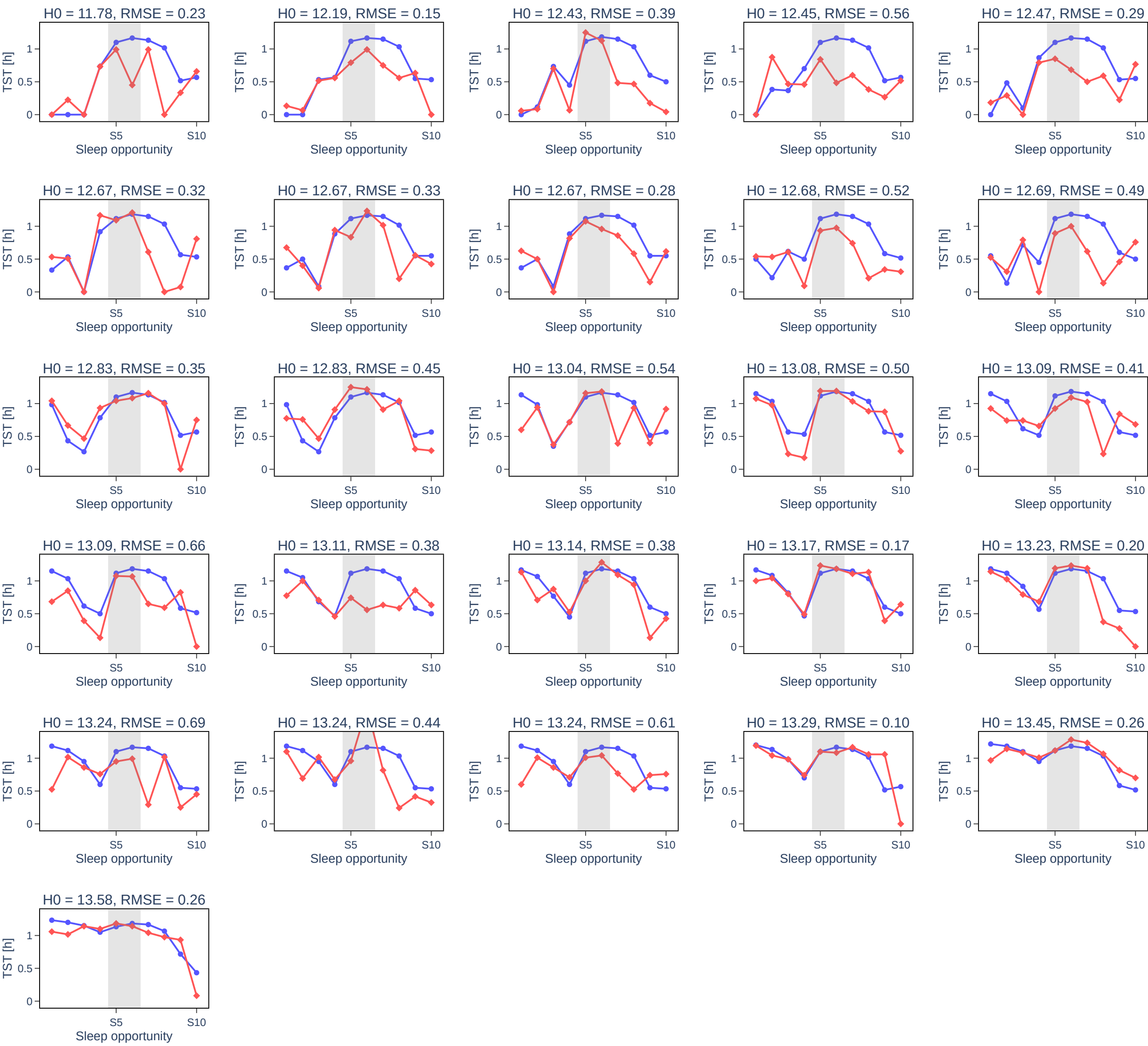

Supplement: Supplementary file 1 [file clockssleep-07-00002-s001.zip › FigureS3_OlderParticipants.pdf]
